# Neurosteroid Binding Sites on the GABA_**A**_ Receptor Complex as Novel Targets for Therapeutics to Reduce Alcohol Abuse and Dependence

**DOI:** 10.1155/2011/926361

**Published:** 2011-10-31

**Authors:** Mary W. Hulin, Russell J. Amato, Johnny R. Porter, Catalin M. Filipeanu, Peter J. Winsauer

**Affiliations:** ^1^Department of Pharmacology and Experimental Therapeutics, LSU Health Sciences Center, 1901 Perdido Street, New Orleans, LA 70112-1393, USA; ^2^Alcohol and Drug Abuse Research Center, LSU Health Sciences Center, New Orleans, LA 70112-1393, USA; ^3^Department of Physiology, LSU Health Sciences Center, New Orleans, LA 70112-1393, USA

## Abstract

Despite the prevalence of alcohol abuse and dependence in the US and Europe, there are only five approved pharmacotherapies for alcohol dependence. Moreover, these pharmacotherapeutic options have limited clinical utility. The purpose of this paper is to present pertinent literature suggesting that both alcohol and the neurosteroids interact at the GABA_A_ receptor complex and that the neurosteroid sites on this receptor complex could serve as new targets for the development of novel therapeutics for alcohol abuse. This paper will also present data collected by our laboratory showing that one neurosteroid in particular, dehydroepiandrosterone (DHEA), decreases ethanol intake in rats under a variety of conditions. In the process, we will also mention relevant studies from the literature suggesting that both particular subtypes and subunits of the GABA_A_ receptor play an important role in mediating the interaction of neurosteroids and ethanol.

## 1. Introduction

The suggestion that neuroactive steroids could have potential as new pharmacotherapies for alcohol abuse and dependence followed shortly after the discovery that ethanol administration released specific neurosteroids. These same data also directly implicated the endogenous neurosteroids as potential contributors to the behavioral effects of ethanol [1, 2]. However, elucidating the interaction between the neuroactive steroids and ethanol has been especially difficult because both produce a wide variety of molecular and behavioral effects and both act at multiple receptors [3, 4]. Complicating matters even further, neurosteroids also have both genomic and nongenomic effects [4] that are often only dissociable in terms of their time course. Thus, the goal of this paper is to present pertinent literature regarding the interaction of ethanol and the neurosteroids while also highlighting research from our laboratory suggesting that one neurosteroid in particular, dehydroepiandrosterone (DHEA), may be a key to discovering promising new therapeutics for treating alcohol abuse and dependence. In this process, we also hope to provide compelling evidence for the involvement of the GABA_A_ receptor complex and the role specific subunits of this complex may play in the effects of DHEA on ethanol intake. 

If there is any doubt that new treatments for alcohol abuse and dependence are needed, one need only to review some of the most recent epidemiological data on excessive alcohol use. In 2009, an estimated 18.6 million persons aged 12 or older met criteria for alcohol dependence or abuse, representing 7.4 percent of the US population [5]. Despite the prevalence of this problem, there are only five approved pharmacotherapies for alcohol dependence in the US and Europe [6]. Furthermore, these pharmacotherapeutic options have limited clinical utility. For instance, the opioid antagonist naltrexone has been shown to have limited success apart from individuals with a family history of alcohol dependence, those with an enhanced opioid response to ingestion of alcohol, those who self-report enhanced alcohol cravings, and individuals with a specific *μ*-opioid receptor polymorphism [7–9]. Acamprosate, a synthetic homotaurine derivative, has been shown to decrease alcohol intake, purportedly via modulation of glutamate [9] and glycine [10] receptors. However, acamprosate had no direct effect on recombinant glutamate or glycine receptors expressed in *Xenopus* oocytes at low, clinically relevant concentrations [11], and therefore, the mechanism by which acamprosate modulates ethanol consumption is still undefined. Experiments involving acamprosate suggest that it is only fully effective in highly motivated subjects with a “goal of abstinence” [12] and that the combined experience of acamprosate with ethanol is necessary for decreasing ethanol intake [13].

## 2. Importance of the GABA_**A**_ System in the Behavioral Effects of Ethanol

Although a variety of neurotransmitters and signaling pathways have been shown to be involved in the behavioral effects of ethanol (e.g., [14–16]), central GABAergic activity is widely accepted to be one of the most important components of ethanol's effects as a CNS depressant [17, 18]. Behaviorally, this supposition is supported by research showing that benzodiazepines and barbiturates that positively modulate the GABA_A_ receptor complex can substitute for ethanol in drug-discrimination procedures [19, 20]. Electrophysiological and genetic techniques have also furthered our understanding of the interaction between ethanol and the GABA_A_ receptor complex by showing that it has both direct and indirect effects on the composition of this heteropentameric chloride ion channel. For example, *in vitro* studies with native and recombinant GABA_A_ receptors indicate ethanol is able to enhance GABA-mediated currents at receptors containing a *δ* subunit (which are found almost exclusively extrasynaptically *in vivo*) and at doses of ethanol consistent with those achieved during typical episodes of social drinking in humans [18, 21, 22]. Studies with mice in which the *δ* subunit has been knocked out have shown the importance of *δ* subunit-containing GABA_A_ receptor complexes in mediating many of the effects of ethanol. These knockout mice are less sensitive to the anticonvulsant effects of ethanol, demonstrate a decreased hyperexcitability during ethanol withdrawal, and show a lower preference for ethanol compared to wild-type controls. In contrast, *δ* subunit knockouts did not differ from controls in ethanol-induced anxiolysis, ataxia, hypnosis, or hypothermia [23]. 

Because *δ* subunits are only found in GABA_A_ receptors that also contain an *α*4 or *α*6 subunit, the importance of these *α* subunit subtypes has been the subject of several investigations and debate. For instance, Hanchar et al. [24] found that cerebellar granule neurons from Sprague-Dawley rats with a naturally occurring mutation in the extrasynaptic *α*6 subunit (arginine (R) to glutamine (Q) in position 100) had an enhanced response to ethanol. Specifically, they reported an increased tonic current amplitude, tonic current noise, and spontaneous inhibitory postsynaptic current. However, using similar methods, Botta et al. [25] found that this mutation did not increase the sensitivity of GABA_A_ receptors to ethanol; rather, they reported that ethanol modulated the currents of these channels indirectly via a presynaptic mechanism. The importance of the *α*4 subunit in mediating the effects of ethanol also remains to be determined, as *α*4 knockout mice had similar anxiolytic, hypothermic, ataxic, and hypnotic responses to ethanol compared to wild-type littermates [26]. 

In addition to modulating GABA_A_ receptors directly, ethanol can also modulate them indirectly by altering the levels of GABA-modulating neurosteroids, such as 3*α*,5*α*-THP (allopregnanolone) and 3*α*,5*α*-THDOC (allotetrahydrodeoxycorticosterone) [1, 2, 27–29]. These neurosteroids are currently thought to contribute to the various behavioral effects of ethanol, including its sedative-hypnotic [30, 31], anxiolytic [32], and discriminative-stimulus effects [33–35]. For example, a reduction in the levels of 3*α*,5*α*-THP and 3*α*,5*α*-THDOC by the 5*α*-reductase inhibitor finasteride blocked the acquisition of ethanol drinking and the development of ethanol preference in male C57BL/6J mice [36]. In healthy, adult social drinkers, finasteride also reportedly decreased the subjective effects of ethanol, leading some investigators to speculate that these neuroactive steroids were integral for producing ethanol's subjective effects [37]. In rats trained to discriminate ethanol from saline, 10 mg/kg of pregnanolone partially substituted (60%–70% drug-lever responding) for the discriminative-stimulus effects of 1 g/kg of ethanol subsequent to chronic administration of either saline or ethanol during adolescence [38]. Similarly, in rats trained to discriminate 5.6 mg/kg of pregnanolone from saline, 1 g/kg of ethanol only partially substituted for this neurosteroid [39]. Together, these symmetrical discrimination data indicate that the neurosteroid pregnanolone has overlapping, but not identical, discriminative-stimulus effects with ethanol. 

In contrast to the partial substitution found with pregnanolone, Gurkovskaya and Winsauer [38] demonstrated that the discriminative-stimulus effects of DHEA, which comes from a common precursor pregnenolone, were unlike those of ethanol in rats trained to discriminate 1 g/kg of ethanol from saline. Furthermore, DHEA only modestly shifted the curve for ethanol-lever responding to the right when it was administered shortly before varying doses of ethanol (0.18–1.8 g/kg). Bienkowski and Kostowski [33] also reported a similar finding in that the sulfated derivative of DHEA, DHEAS, was ineffective at blocking the discriminative-stimulus effects of ethanol. Thus, the effects of DHEA on the discriminative-stimulus effects of ethanol are similar to those of RO15-4513, a partial inverse agonist at the benzodiazepine receptor site, which negatively modulates the GABA_A_ receptor complex and has only been shown to modestly attenuate the subjective effects of ethanol (for review, see [14]). When these data are considered together, there seems to be little evidence to suggest that compounds that negatively modulate the GABA_A_ receptor alter the discriminative-stimulus effects of ethanol even though these drugs can attenuate some of the other behavioral effects of ethanol. 

Another mechanism by which ethanol enhances GABAergic activity indirectly is by increasing presynaptic GABA release [40–42]. Roberto et al. [42] found that direct infusion of 44 mM ethanol to slices of neurons from the central amygdala of rats reduced paired-pulse facilitation and increased the frequency of spontaneous inhibitory post-synaptic potentials and currents (IPSP/IPSCs), changes that the investigators concluded were indicative of increased presynaptic GABA release. In addition, studies have shown increases in the frequency of miniature ISPCs with 100 mM ethanol in Golgi cells from rat cerebellar slices and 70 mM ethanol in spinal motor neurons [40, 41]. 

Just as GABAergic activity contributes to ethanol's CNS depressant and discriminative-stimulus effects, it is also thought to be integrally involved in mediating the reinforcing effects of ethanol. This notion has been strongly supported by studies showing that decreases in GABA_A_ receptor activity can decrease ethanol intake. More specifically, GABA_A_ receptor antagonists as well as inverse agonists at the benzodiazepine receptor site have been shown to decrease both ethanol preference and operant responding for ethanol [43–47]. For example, injection of 2 ng of the competitive GABA_A_ receptor antagonist SR 95531 directly into the central nucleus of the amygdala decreased operant responding for ethanol in male rats, demonstrating a direct link between GABA_A_ receptor modulation and the reinforcing effects of ethanol [48]. These results were similar to those with RO15-4513 [49], which has been shown to decrease ethanol intake and to antagonize the intoxicating effects of ethanol [45, 50–52] when administered under an operant schedule of food- and ethanol-reinforced responding. In addition, RO15-4513 has been shown to reverse the memory-impairing effects of ethanol [53–55], and this reversal was attributed to both compounds' interaction with ethanol at the GABA_A_ receptor.

Although ethanol's effects on the brain are pervasive, the role of GABA and dopamine in parts of the mesolimbic dopamine system, such as the ventral tegmental area, central nucleus of the amygdala, and nucleus accumbens, are of particular interest with regard to the reinforcing effects of ethanol. Ikemoto et al. [56] found that dopaminergic neurons in the anterior and posterior portions of the ventral tegmental area are differentially regulated by GABA_A_ receptor modulators, as evidenced by a series of studies in which rats self-infused the GABA_A_ receptor antagonist picrotoxin into the anterior, but not posterior, ventral tegmental area. Conversely, rats self-infused the GABA_A_ receptor agonist muscimol into the posterior, but not anterior, ventral tegmental area [56, 57]. This was further clarified in a microdialysis study by Ding et al. [58], in which they found that the anterior ventral tegmental area was predominantly under GABA-mediated tonic inhibitory control, whereas the posterior tegmental area was predominantly under the control of dopamine-mediated inhibition. These data, therefore, suggested that the posterior ventral tegmental area may be of more importance than the anterior ventral tegmental area in the reinforcing effects of ethanol. The ventral tegmental area has direct projections to the nucleus accumbens, an area of the brain classically associated with the translation of “motivation to action,” or a link between areas of the brain associated with reward and those associated with drug seeking [59]. Furthermore, ethanol consumption in alcohol-preferring rats has been shown to increase extracellular dopamine content in the nucleus accumbens [60].

## 3. Effects of DHEA on GABA_**A**_ Receptors

The discovery of steroid synthesis in the brain quickly resulted in numerous studies into the physiological roles of these “neurosteroids,” with an emphasis on their apparent nongenomic effects [29, 61–64]. In 1990, Majewska et al. [65] demonstrated that the sulfated form of DHEA (DHEAS) bound to the GABA_A_ receptor on rat neurosynaptosomes. Further, they showed that DHEAS binding decreased GABA-mediated current using a whole-cell voltage-clamp technique. Le Foll et al. [66] confirmed these findings using a whole-cell voltage-clamp technique in frog pituitary cells and also determined that 10 *μ*M of DHEA and DHEAS were equally effective at decreasing GABA-induced currents. The next year, Imamura and Prasad investigated the effects of DHEA and DHEAS on GABA-mediated chloride influx in neurosynaptosomes derived from rat cortex, hippocampus, and cerebellum. These investigators determined the effects of multiple concentrations of DHEA and DHEAS on GABA-mediated chloride influx and concluded that DHEAS altered chloride influx with greater potency than DHEA [67]. Park-Chung et al. [68] also found a difference in potency between DHEA and DHEAS, as 100 *μ*M DHEAS was nearly twice as effective at decreasing GABA-induced current as an equal concentration of DHEA in *Xenopus* embryos expressing *α*1*β*2*γ*2 GABA_A_ receptors. 

Because multiple studies have shown that DHEAS is more potent than DHEA, the binding characteristics of DHEAS at the GABA_A_ receptor have been more widely studied and characterized. Studies that have investigated the putative binding sites for DHEAS suggest that neurosteroids that are negative modulators of the GABA_A_ receptor complex act at sites distinct from those that are positive modulators. For some neurosteroids, such as pregnanolone, the addition of a negatively charged sulfate group changes the GABA-modulating capacity of the neurosteroid from positive to negative. Substitution of a hemisuccinate group for the sulfate imparts the same effect on modulator activity, indicating that the negative charge of the compound influences its activity [68]. These data support the suggestion that sulfated and unsulfated steroids modulate GABA_A_ receptor activity through different sites [68, 69]. Unfortunately, similarities between DHEAS and DHEA binding are unknown, and additional research will be necessary to clarify these issues. 

The binding sites for neurosteroids that positively modulate the GABA_A_ receptor complex, such as 3*α*,5*α*-THP and THDOC, have been more thoroughly investigated [62, 70, 71]. The results from these studies have indicated that these steroids act at one of two putative neurosteroid binding sites. The first site is thought to reside within the transmembrane domains of the *α* and *β* subunit interface, whereas the second site is thought to reside on the *α* subunit (for review, see [70]). The failure of DHEA to attenuate the pregnanolone-induced disruptions in behavior maintained under a differential-reinforcement-of-low-rate schedule [72] suggests that DHEA binds to a site on the GABA_A_ receptor separate from pregnanolone. Therefore, DHEA is suspected to act at a site on the GABA_A_ receptor distinct from the binding site of sulfated neurosteroids such as DHEAS and from neurosteroids that are positive modulators of the GABA_A_ receptor such as pregnanolone. 

**Figure 1:**
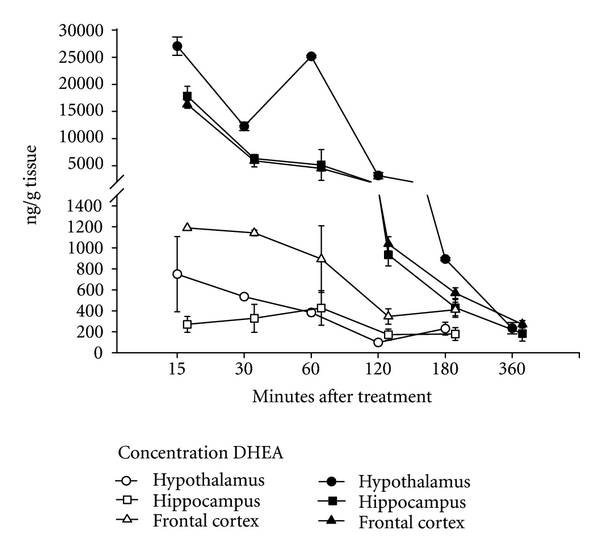
The amount of DHEA (ng/g) present in the hypothalamus, hippocampus, and frontal cortex of rats over a six-hour time period after a single acute intraperitoneal injection. Adult male Long-Evans rats received either 56 mg/kg of DHEA (*n* = 6) or an equal volume of cyclodextrin vehicle (*n* = 5). A DHEA-treated subject was sacrificed with a vehicle-treated control at 15, 30, 60, 120, and 180 minutes after injection, while the final DHEA-treated subject was sacrificed 360 minutes after injection. Brains were collected, flash frozen, and later dissected using the Glowinski technique [75]. Steroids were extracted from the hypothalamus, hippocampus, and frontal cortex of each subject using the solid-phase extraction method described and validated by Newman et al. [74]. Briefly, tissue from each region was prepared in an aqueous matrix and steroids were extracted from each sample using a C18 column primed with ethanol and equilibrated with water. Each sample was eluted, dried, and resuspended in deionized water, and DHEA levels were determined using ELISA.

Despite the differences in potency between DHEA and DHEAS, DHEA may have greater clinical utility because of its capacity to cross the blood-brain barrier. The sulfate group of DHEAS imparts greater hydrophilicity to the compound, largely limiting its capacity for diffusing into the central nervous system without first being hydrolyzed to the free steroid [73]. The more lipophilic DHEA crosses the blood-brain barrier in large amounts, as evidenced by recent work in this laboratory. In this experiment, adult male Long-Evans rats were administered 56 mg/kg of DHEA and then sacrificed along with vehicle-treated control rats at time points ranging from 15 minutes to six hours after injection for brain steroid analysis. Steroids were extracted using the solid-phase technique established and validated by Newman et al. [74] and then analyzed using a commercially-available ELISA (DHEA Saliva ELISA kit, IBL International, Hamburg, Germany). As shown in Figure 1, DHEA levels in the hippocampus, hypothalamus, and frontal cortex of the brain were over twentyfold greater than in vehicle-treated controls. In fact, fifteen minutes following intraperitoneal (i.p.) injection, DHEA was present at concentrations shown by Majewska [61] to negatively modulate the GABA_A_ receptor. 

General support for the behavioral effects of DHEA as a negative modulator of the GABA_A_ receptor complex comes from a study by Amato et al. [72], who demonstrated that the acute effects of DHEA administration on behavior were similar to other negative or neutral GABA_A_ modulators under a differential-reinforcement-of-low-rates (DRL) schedule in rats. This study compared a variety of positive modulators to DHEA and the negative modulator *β*-CCM and the neutral modulator flumazenil across several dependent measures. Interestingly, DHEA was similar to *β*-CCM and flumazenil in producing little or no effect on response rate or the temporal pattern of responding. These findings directly contrast with the effects of the positive modulators ethanol, pregnanolone, lorazepam, and pentobarbital on behavior maintained under the same schedule, as these drugs increased response rate and disrupted the temporal pattern of responding. 

The negative modulators of the GABA_A_ receptor complex also contrast with the positive modulators in terms of their effects on anxiety. For instance, the benzodiazepines and barbiturates that positively modulate the GABA_A_ receptor complex typically decrease anxiety in animal models as indicated by increases in suppressed behavior [78–80], time spent in open arms of the elevated plus maze [81, 82], and exploration in the open field test [83]. In humans, benzodiazepines are prescribed clinically as anxiolytics. Unlike these drugs, the negative modulators such as the beta carbolines that are inverse agonists at the benzodiazepine binding site are anxiogenic [82]. While one would expect all negative modulators of the GABA_A_ receptor complex to be anxiogenic, this does not seem to be the case for DHEA as several studies have demonstrated that DHEA is anxiolytic in situations involving chronic stress [84–86]. Presumably, the anxiolytic effects of DHEA can be attributed to its antiglucocorticoid properties, especially considering most subjects under chronic stress have increased levels of cortisol. The DHEA/cortisol ratio is of particular significance, as the antiglucocorticoid effects of DHEA are postulated to be the means by which DHEA was able to reduce depression in humans [87, 88]. Charney [84] has also suggested that DHEA may be valuable for reducing the response to stress, particularly in patients with post-traumatic stress disorder. 

In addition to the antiglucocorticoid effects of DHEA, the capacity of DHEA to modulate the release of other GABA-modulating neurosteroids may also be involved in its anxiolytic effects. DHEA administration has been shown to increase peripheral levels of 3*α*,5*α*-THP in postmenopausal women that received 25 mg/day for three months [89] and both peripheral and CNS levels of 3*α*,5*α*-THP in female rats that received 2 mg/kg for 14 consecutive days [90]. Together with the finding that two weeks of DHEA administration decreased central levels of pregnenolone sulfate [91], another neurosteroid that negatively modulates the GABA_A_ receptor [64, 66], these data suggest that long-term DHEA administration may increase overall GABAergic tone despite its capacity for negatively modulating the GABA_A_ receptor complex acutely. Whether this is a direct effect of DHEA or a compensatory response to chronic DHEA remains an important question that will require further investigation. 

As mentioned previously, the five subunits that comprise the GABA_A_ receptor complex affect the responsiveness of these receptors to various endogenous and exogenous substances such as the neurosteroids and benzodiazepines [71, 92, 93]. Moreover, the repeated stimulation of GABA_A_ receptor subtypes can induce changes in the subunits comprising these receptors. The *α*4 subunit, for example, has been shown to be particularly sensitive to changing levels of neurosteroids [93] and was upregulated following chronic administration of progesterone (a precursor to 3*α*,5*α*-THP) and following withdrawal of progesterone treatment. This particular subunit is also of interest because it was upregulated following chronic treatment with benzodiazepines, and its expression decreased the sensitivity of the GABA_A_ receptor complex to benzodiazepines [94]. For this reason, GABA_A_ receptors containing an *α*4 subunit are often referred to as “benzodiazepine insensitive” receptors. The capacity of GABA_A_ ligands to modify GABA_A_ receptor subunit expression is, therefore, another putative mechanism by which DHEA treatment might alter ethanol intake and preference. 

**Figure 2:**
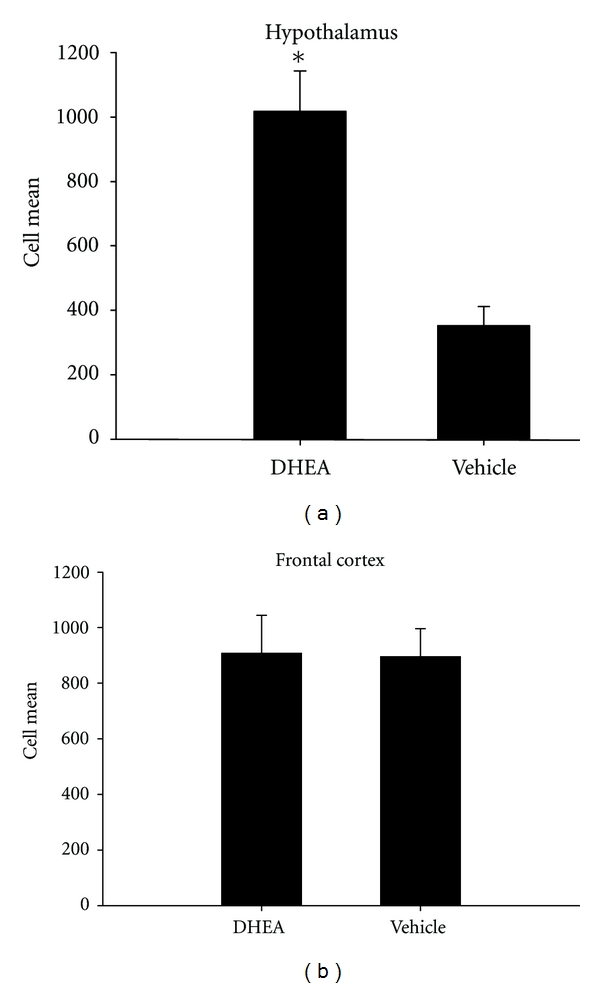
Mean number of GABA_A_ alpha-4 subunit transcript copies per cell in the hypothalamus (top panel) and frontal cortex (bottom panel) of rats administered 56 mg/kg of DHEA or vehicle. Drug-naïve male rats received either DHEA (*n* = 12) or an equal volume of cyclodextrin vehicle (*n* = 12) for ten consecutive days; on the tenth day, subjects were sacrificed and their brains were collected. Brains were dissected using the Glowinski technique [75], and each brain region was pooled and homogenized. Due to the high lipid content of the samples, a spin column technique was utilized for the RNA extraction. RNA analysis was performed using TaqMan assay kits (Applied Biosystems, Foster City, Calif, USA). Approximately 1 to 2 *μ*L of each sample were used to determine the RNA concentration in each sample using Nanodrop. Values are expressed as a fraction of a normalizing gene, ribosomal 18S RNA.

**Figure 3:**
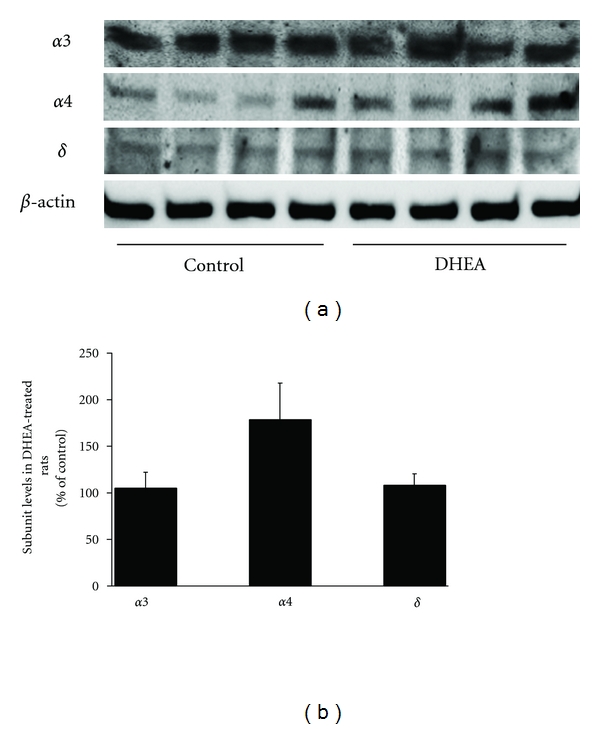
Expression of the *α*4, *α*3, and *δ* subunits of GABA_A_ receptors in the hypothalamus of rats administered 56 mg/kg of DHEA for 10 consecutive days as measured by Western blot analysis. 100 *μ*g of tissue from each area was resuspended in lysis buffer (20 mM Tris pH 8.0, 137 mM NaCl, 0.5 mM sodium orthovanadate, 2 mM okadaic acid, 10% glycerol, 1% Nonidet P40, 2% protease inhibitor) and processed for protein extraction using MicroRoto for Lysis Kit (Bio-Rad, Hercules, Calif, USA). The Bradford Method [76] was used to determine protein concentration, and then samples were diluted, separated by SDS-PAGE, and transferred to nitrocellulose PDVF membranes (Amersham Biosciences, Piscataway, NJ, USA). The membranes were immunoblotted for two hours at room temperature with two specific antibodies, a rabbit anti-*α*4 antibody at a 1 : 500 dilution (Santa Cruz Biotechnology, Santa Cruz, Calif, USA), and a mouse anti-*β*-actin diluted in a proportion of 1 : 2000 (Santa Cruz Biotechnology). A specific secondary antibody (PerkinElmer Life Sciences, Waltham, Mass, USA) followed at a dilution of 1 : 2000. Expression was visualized using ECL Plus (PerkinElmer) and a Fuji Film luminescent image analyzer (LAS-1000 Plus, Fuji Photo Film Co. Ltd., Tokyo, Japan). The images were then quantified by densitometry using the Image Gauge program [77], and the expression value of each subunit was normalized to *β*-actin values.

This notion led us to investigate the effect of DHEA administration on the expression of the *α*4 subunit of the GABA_A_ receptor complex. In this study, twenty-four drug-naïve male Long-Evans hooded rats received either 56 mg/kg of DHEA (*n* = 12) or vehicle (*n* = 12) daily for a ten-day period. On the final day of treatment, subjects were sacrificed and the brains collected for analysis. Quantitative analysis of mRNA transcripts indicated that DHEA-treated rats had an approximately threefold increase in expression of *α*4 subunit mRNA in the hypothalamus compared to vehicle-treated controls, as shown in Figure 2. Interestingly, the expression of the *α*4 subunit mRNA in the frontal cortex did not differ between treatment groups. Together, these data suggested that the capacity of DHEA to alter *α*4 subunit expression is brain-region dependent, and this was further supported by Western-blot analysis showing that *α*4 subunit protein expression was increased in the hypothalamus following DHEA treatment compared to control (see Figure 3). In addition, expression of the *δ* subunit, which is expressed nearly exclusively in receptor complexes with either the *α*4 or *α*6 subunits, was not altered by DHEA treatment. More studies are certainly warranted to determine the implication of these findings.

## 4. DHEA Decreases Ethanol Intake

Working under the hypothesis that negative modulators of the GABA_A_ receptor complex generally decrease ethanol intake, we initiated a series of studies to determine if DHEA could produce the same effect. Using a relatively standard ethanol preference procedure, our first study compared the effects of DHEA and pregnanolone on home-cage ethanol intake and found that DHEA was more effective at reducing the intake of an 18% (v/v) ethanol solution than pregnanolone [95], which has been shown to positively modulate the GABA_A_ receptor complex. These results were important for several reasons. First, they showed that the neurosteroids remain a relatively unexplored class of drugs with enormous therapeutic potential. Second, they showed that neurosteroids with the capacity to negatively modulate the GABA_A_ receptor complex may be as valuable, or more valuable, as therapeutics for alcohol abuse and dependence than positive modulators, which could putatively serve as substitution therapies for alcohol. An important methodological detail in this study was that neurosteroid injections were administered daily until a criterion for stable ethanol intake was achieved; namely, each dose of neurosteroid was administered until ethanol intake did not vary by more than ±20% for 3 days or for a total of 8 days, in which case the last 3 of those 8 days were used for comparison purposes. This criterion was largely instituted because (1) the intake of low concentrations of ethanol (or low doses of other self-administered drugs) is inherently variable, (2) acute administration of a potential therapeutic may not always be representative of a drug's capacity to reduce self-administration, and (3) therapeutics for drug dependence are generally administered chronically as opposed to acutely. However, using this criterion raised several critical questions regarding DHEA's mechanism of action. First, were multiple injections necessary to achieve the effect on ethanol intake, and second, were the decreases in ethanol intake an effect of DHEA or one of several metabolites including the sex hormones testosterone and estradiol? 

To address these questions, Worrel et al. [96] administered 7-keto DHEA, a metabolite of DHEA that is not metabolized to testosterone or estradiol [97], to the subjects from the Gurkovskaya et al. [95] preference study and found that this compound produced effects as large as DHEA. In fact, while 10 mg/kg of 7-keto DHEA produced an effect comparable to DHEA, 56 mg/kg of 7-keto DHEA produced a larger decrease in ethanol intake than DHEA. Another important aspect of this study was that 7-keto DHEA decreased ethanol intake after the initial injection, which occurred 15 minutes prior to the 30-minute preference session when ethanol and water were presented. Together, these data indicated that a major metabolite of DHEA might be responsible for the effect of DHEA, metabolism of DHEA to the sex hormones was not necessary to produce an effect on ethanol intake, and repeated administration was not necessary to produce an effect with 7-keto DHEA. Given the onset of the effect of 7-keto DHEA on ethanol intake, these data also suggest that a nonsteroidal, rather than steroidal, mechanism of action might be responsible for DHEA's observed effects. 

**Figure 4:**
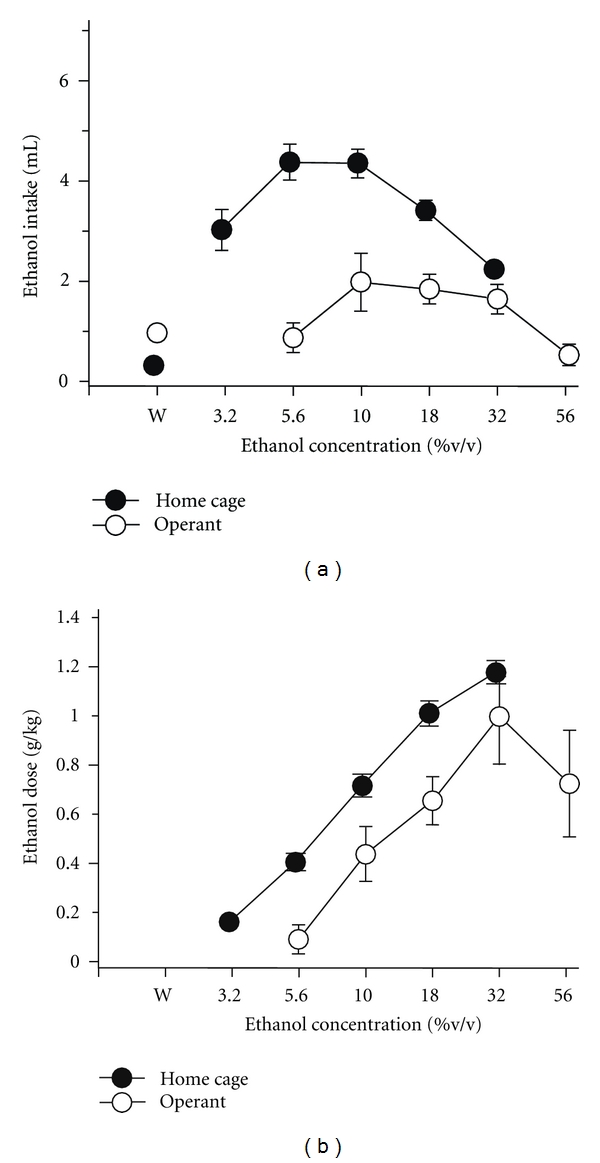
Effects of ethanol concentration on ethanol intake (mL) and the dose of ethanol (g/kg) consumed under home cage (*n* = 22) and operant (*n* = 5) self-administration procedures. Filled circles represent voluntary home cage ethanol intake, whereas unfilled circles represent operant ethanol intake under a fixed ratio (FR) 10 schedule of reinforcement. The points and vertical lines above “W” indicate the means ± standard error of the mean (SEM) for sessions in which water was available (control). The points with vertical lines in the concentration-effect data indicate the mean ± SEM for each ethanol concentration. The points without vertical lines indicate instances in which the SEM is encompassed by the point.

**Figure 5:**
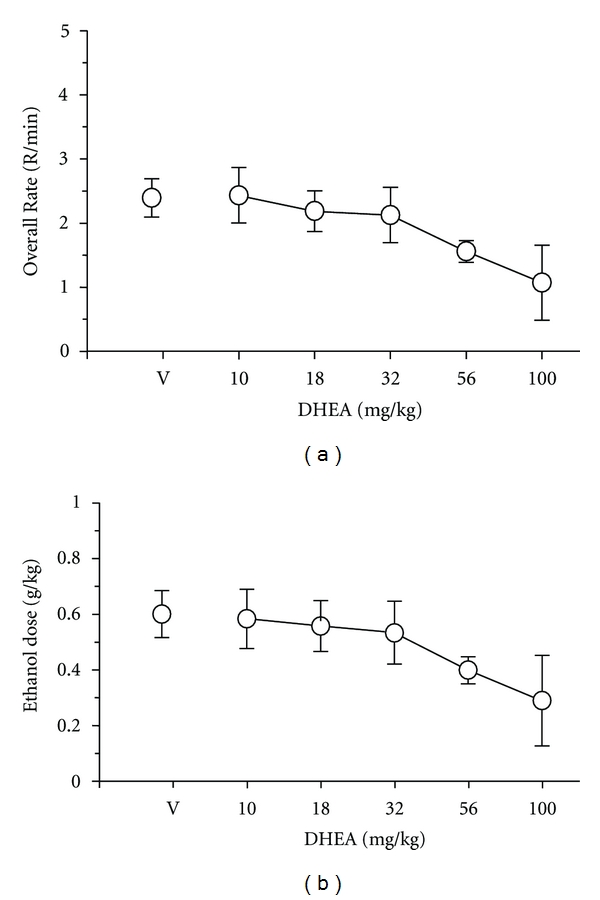
Effects of intraperitoneal administration of DHEA on rats (*n* = 5) responding under an FR-10 schedule for 0.1 mL of 18% (v/v) ethanol. The dependent measures were response rate in responses/min and the dose of ethanol presented in g/kg. The points and vertical lines above “V” indicate the means and standard error of the mean (SEM) for sessions in which vehicle was administered (control). The points with vertical lines in the dose-effect data indicate the mean ± SEM for sessions in which DHEA was administered. The points without vertical lines indicate instances in which the SEM is encompassed by the point.

In more recent studies conducted in our laboratory, we have established ethanol self-administration under an operant schedule of reinforcement in order to compare the effects of DHEA (and 7-keto DHEA) on voluntary versus schedule-controlled ethanol intake. To establish ethanol-maintained behavior, rats were trained to respond under a fixed-ratio 10 schedule in which every 10 responses on a lever dispensed 0.1 mL of 18% ethanol to a concave spout located on the front wall of an operant chamber. After ethanol intake stabilized under these contingencies, the substitution of different ethanol concentrations was undertaken to compare the concentration-effect curve for ethanol under the FR-10 schedule with the curve established under the home-cage preference procedures. Interestingly, although intake of the lower concentrations of ethanol was more robust under the home-cage preference procedure than the operant procedure, the intake and dose of ethanol between the two procedures was more similar for the higher ethanol concentrations. In particular, substitution of a 32% ethanol concentration for the 18% ethanol concentration produced similar intake in milliliters and in the dose consumed (see Figure 4). More important, doses of DHEA that decreased ethanol intake under the home-cage preference procedure also decreased ethanol intake under an operant procedure (e.g., 56 mg/kg; Figure 5). 

As a means of showing the potential developmental influence of DHEA on ethanol preference, we administered DHEA, lorazepam, or vehicle to three groups of male rats during adolescence and then assessed preference and intake of ethanol during adulthood [98]. Lorazepam was included specifically as a comparison to DHEA, because it is well known as a positive allosteric modulator of the GABA_A_ receptor complex. Briefly, each group of adolescent rats received a total of 15 injections (12 of one dose and 3 of a higher dose) on postnatal days (PND) 35–64, and then after a period of no treatment received 23-hours access to water, saccharin, or an ethanol/saccharin solution over several days on two separate occasions (PND 88 and again at PND 111). On the last occasion, the concentration of ethanol in the ethanol/saccharin solution was also increased to determine if the adolescent treatments altered the concentration-effect curves for each group. In general, this study demonstrated that lorazepam administration during adolescence increased adult preference for ethanol compared to vehicle or DHEA administration, whereas DHEA decreased adult preference for ethanol and saccharin compared to vehicle administration. These data were remarkable not only because they showed the long-term effect of positive allosteric modulation of GABA_A_ receptors on later, adult ethanol intake, but they suggest the potential for endogenous levels of DHEA to play an integral role in shaping adult preference and intake either through its putative effects on the GABA_A_ receptor complex or through other as yet unknown mechanisms [98].

## 5. Summary and Conclusions

Although the exact mechanism by which DHEA decreases ethanol intake is still under investigation, studies from both the literature and our laboratory strongly indicate that it can interact both directly and indirectly with the GABA_A_ receptor complex and that its behavioral effects are very similar to those of several other negative GABA_A_ receptor modulators. Consistent with data generated over the past several years [21, 22], our data also emphasize the potential role of extrasynaptic GABA_A_ receptors in the interaction of the neurosteroid DHEA and alcohol. For instance, recent electrophysiological and biochemical data have indicated that GABA_A_ receptors containing a *δ* subunit are potently affected by both ethanol and neurosteroids and that these “extrasynaptic” receptors likely contribute to tonic IPSP and IPSCs in many brain regions. Furthermore, GABA_A_ receptors with *δ* subunits are thought to be associated exclusively with *α*4 and *α*6 subunits *in vivo*. If this is the case, the upregulation of the *α*4 subunit could then affect the responsiveness of *δ*-containing GABA_A_ receptors, and ultimately, the behavioral effects of ethanol or the neurosteroids. 

Similar to our molecular data pointing to a DHEA-ethanol interaction, our behavioral studies show that DHEA can dose dependently decrease ethanol intake in outbred rats. Interestingly, some of these data were gathered prior to definitively knowing whether peripherally administered DHEA crossed the blood brain barrier and whether DHEA or one of its hormonal metabolites was responsible for the effect. Since then, however, we have conducted studies showing that DHEA readily crosses the blood brain barrier after peripheral administration (data shown above) and that metabolism of DHEA to one of the sex hormones (i.e., either testosterone or estradiol) is not necessary to obtain the decrease in ethanol intake [96]. Moreover, we have shown DHEA can decrease ethanol intake that is voluntary [95] or controlled by an operant schedule of reinforcement. 

Unfortunately, the effect of DHEA on ethanol intake cannot be attributed exclusively to its capacity for negatively modulating GABA_A_ receptor though there is a significant amount of data showing that this capacity may be its most prominent nongenomic effect [65, 67]. Without question, the difficulty identifying a binding site for DHEA on the GABA_A_ receptor complex has made the investigation into DHEA's mechanism of action more problematic. As indicated in this paper, the binding site for DHEA would seem to be different from the site for sulfated neurosteroids [68, 69] and from the site for positive GABA_A_ modulators [70]. From a behavioral perspective, however, DHEA produces effects similar to other negative modulators in rats responding under at least one operant schedule of reinforcement (i.e., a DRL schedule). The most notable exception to DHEA's profile as a negative modulator seems to be its capacity for producing anxiolytic, rather than axiogenic, effects [84–86]. This could be viewed as a therapeutic benefit for a medication that is used to treat alcohol abuse and dependence. Furthermore, unlike negative modulators such as RO15-4513, there is very little evidence that DHEA or 7-keto DHEA have proconvulsant effects [49, 99]. By contrast, numerous small clinical trials with DHEA have shown adverse effects predominantly related to the androgenic effects of DHEA [100, 101]. For instance, common adverse effects in women taking 200 mg of DHEA per day include acne and hirsutism. These effects may be averted, however, by administering 7-keto DHEA, which is not converted to sex hormones [97] and reduces ethanol intake similarly to DHEA [96].
